# Complementing tissue characterization by integrating transcriptome profiling from the Human Protein Atlas and from the FANTOM5 consortium

**DOI:** 10.1093/nar/gkv608

**Published:** 2015-06-27

**Authors:** Nancy Yiu-Lin Yu, Björn M. Hallström, Linn Fagerberg, Fredrik Ponten, Hideya Kawaji, Piero Carninci, Alistair R. R. Forrest, The FANTOM Consortium, Yoshihide Hayashizaki, Mathias Uhlén, Carsten O. Daub

**Affiliations:** 1 Department of Biosciences and Nutrition, Karolinska Institute, Huddinge, 14183, Sweden; 2 Science for Life Laboratory, Karolinska Institute, Solna, 17121, Sweden; 3 Science for Life Laboratory, KTH-Royal Institute of Technology, Solna, 17121, Sweden; 4 Department of Immunology, Genetics and Pathology, Science for Life Laboratory, Uppsala University, Uppsala, 751 85, Sweden; 5 RIKEN Preventive Medicine and Diagnosis Innovation Program, Wako, Saitama 351-0198, Japan; 6 RIKEN Center for Life Science Technologies (CLST), Division of Genomic Technologies, RIKEN Yokohama Institute, Tsurumi-ku, Yokohama, 230-0045, Japan; 7 RIKEN Omics Science Center1^‡^, Yokohama, Kanagawa, 230-0045, Japan

## Abstract

Understanding the normal state of human tissue transcriptome profiles is essential for recognizing tissue disease states and identifying disease markers. Recently, the Human Protein Atlas and the FANTOM5 consortium have each published extensive transcriptome data for human samples using Illumina-sequenced RNA-Seq and Heliscope-sequenced CAGE. Here, we report on the first large-scale complex tissue transcriptome comparison between full-length versus 5′-capped mRNA sequencing data. Overall gene expression correlation was high between the 22 corresponding tissues analyzed (*R* > 0.8). For genes ubiquitously expressed across all tissues, the two data sets showed high genome-wide correlation (91% agreement), with differences observed for a small number of individual genes indicating the need to update their gene models. Among the identified single-tissue enriched genes, up to 75% showed consensus of 7-fold enrichment in the same tissue in both methods, while another 17% exhibited multiple tissue enrichment and/or high expression variety in the other data set, likely dependent on the cell type proportions included in each tissue sample. Our results show that RNA-Seq and CAGE tissue transcriptome data sets are highly complementary for improving gene model annotations and highlight biological complexities within tissue transcriptomes. Furthermore, integration with image-based protein expression data is highly advantageous for understanding expression specificities for many genes.

## INTRODUCTION

Gene expression diversity within the different body parts of a living organism contributes to the distinct phenotypes, physiology and functionalities of the different cell types and tissues. Knowing the gene expression profiles of all the major tissues in the human body can greatly increase our understanding of human biology, aid with disease state diagnosis, and help identify potential drug target candidates. With regards to human biology, fundamental questions in terms of gene expression include: which genes are expressed in all tissues/cell types, and which combination of genes uniquely gives an organ its tissue identity. Whether a gene is truly tissue-specific or is found in a number of distinct tissues can only be assessed if its expression has been profiled in most parts of the human body.

Many genome-wide transcriptome profiling technologies have been developed for identification and quantification of global gene expressions, each emphasizing specific aspects of the RNA transcripts. For example, microarrays were developed to measure relative quantity of RNA expression for comparing treatment to control samples (1). As sequencing technologies improved drastically, RNA-Seq was developed to provide identification as well as quantification of RNA transcripts (2). Developed by RIKEN in Japan, the cap analysis gene expression (CAGE) technology focuses on defining the promoter landscape, which is important for understanding gene regulation by transcription factors and enhancers (3–5).

As the technologies matured, many efforts have been made to gather gene expression profiles for various parts of the human body, using the different technologies mentioned above. One of the first efforts to put together a comprehensive tissue transcriptome microarray profile (6) is now accessible through the bioGPS portal (7), among other data integration websites. General repositories of microarray data from tissues can be found at ArrayExpress (8). Examples of Next-generation sequencing (NGS) RNA-Seq tissue transcriptome data include the Illumina BodyMap 2.0, which has been incorporated into GeneCards (9) and other resources; RNA-Seq Atlas, which holds data for 11 healthy tissues, is cross-linked with microarray data for both healthy and pathological patient samples (10). The Genotype Tissue Expression Project (GTEx), which focuses on identifying expression quantitative trait locus (eQTL) data, aims to collect tissue data from 900 individuals by 2015 (11). As a public data repository, Expression Atlas (12) provides differential expression data for both microarrays and NGS sequencing produced by different labs under many experimental conditions.

Recently, the Human Protein Atlas (HPA), a resource that provides immunohistochemistry-based expression, spatial localization within tissues, and subcellular localization information for the human proteome, has produced a large tissue RNA-Seq expression repository, with 32 histologically normal human tissues based on 95 individuals, with at least 2 biological replicates for each tissue (13). The FANTOM5 (Functional Analysis of Mammalian Genomes 5) consortium has also recently published transcriptome profiles for over 975 human samples, including cell line, primary cells, and tissues using CAGE with single-molecule sequencing technology (14,15). Both of these projects employed systematic laboratory workflows for preparing RNA from samples, producing transcriptome sequencing results, as well as coherent computational pipelines for data processing.

**Figure 1.**
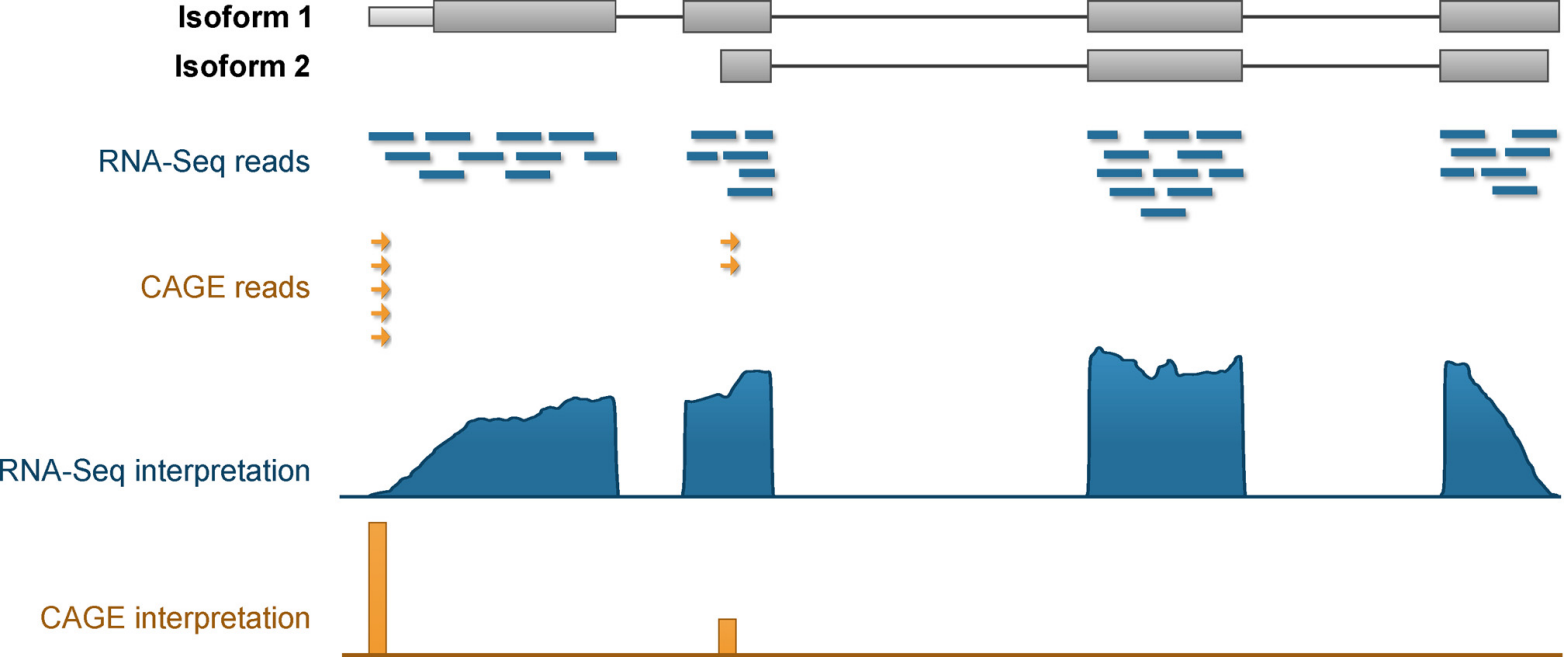
Schematic diagram of CAGE and RNA-Seq read coverage for a gene with two isoforms. RNA-Seq reads are 100 bp short reads that cover the entire transcript with decreasing coverage at the 5′ and 3′ ends of the transcripts, while CAGE reads (∼27 bp) provide sharp coverage for transcript start sites (TSS) of the first exons for all transcripts that are expressed.

While both data sets contain measurements for RNA transcription levels, the FANTOM5 CAGE technology is distinct from RNA-Seq in terms of the part of RNA captured (Figure 1). CAGE technology captures the capped 5′ start of mRNA and sequences around 27 base pairs, whereas RNA-Seq technology sequences the entire length of transcripts covering all the exons. Whereas the RNA-seq protocol used by the HPA project captures only polyadenylated transcripts, the protocol of CAGE employed does not suffer from this limitation, and will detect all 5′-capped transcripts, regardless of polyadenylation state. One of the biggest strengths of deep CAGE sequencing lies in its ability to distinguish closely spaced transcript start site (TSS) usage preference and the corresponding promoter landscapes. The HeliScope sequencer used to generate the FANTOM5 CAGE data does not require any polymerase chain reaction (PCR) steps and provides PCR-bias free sequencing data. On the other hand, RNA-Seq provides clear information for exons that are present in the sample and can infer which transcript isoforms are preferentially expressed.

It has been shown that clonally amplified Illumina sequencing and single molecule HeliScope sequencing transcriptome profiling are technically comparable when a limited number of cell line RNAs were profiled by both techniques in the same lab (16). However, it is not known to what extent complex tissue gene expression data is directly comparable, especially if tissues are obtained from different sources and processed in different manners. In this study, we performed global comparisons for twenty-two tissues between the FANTOM5 CAGE and HPA RNA-Seq data sets. We compared the data from the tissue perspective, looking at gene expression correlation between two corresponding tissues. We evaluated global comparisons of all tissues against all across two data sets. We also compared the data from the perspective of gene categories to see how ubiquitously expressed genes and single-tissue enriched genes (expressed considerably higher in one tissue than all other tissues) compare between the two data sets. Finally we looked at disagreements between the two data sets and provide some explanations for these discrepancies.

This work is part of the FANTOM5 project. Data download, genomic tools and co-published manuscripts have been summarized at http://fantom.gsc.riken.jp/5/.

## MATERIALS AND METHODS

### Data set selection

The HPA RNA-Seq data was obtained from Array Express Archive (www.ebi.ac.uk/arrayexpress/) with the accession number: E-MTAB-1733 (17). CAGE peaks expression table was obtained from http://fantom.gsc.riken.jp/5/data (15). The ‘robust’ promoter set was used for this study. Out of 27 tissues with available RNA-Seq data from the HPA data set, only tissues with corresponding FANTOM5 tissues were selected for this study. Adult duodenum and stomach tissues were not available in the FANTOM5 data set. The skin, bone marrow, and adrenal gland FANTOM5 samples were not used in this study due to low total TPM sums. 22 tissues passed the selection criteria—including 27 samples from FANTOM5, as well as 79 samples from HPA—were used in this study. The full list of tissues used in this study is available in Supplementary Table S1.

### Gene annotation

RNA-Seq data was annotated as previously described (17), using GRCh37 as reference genome for mapping reads with Tophat v2.0.3 (18) and using the gene annotation from Ensembl build 73 (19) for expression quantification (in FPKMs) with Cufflinks v2.0.2 (20). CAGE clusters were assigned to genes with a custom script using the same genome build and Ensembl annotation as for RNA-Seq data. Peaks mapped to within ±500 bases of any annotated transcript start sites were summed up for each of the associated gene. Both protein-coding and non-coding genes were used for the initial overall comparisons, but only genes annotated as protein-coding (20,940 in total) were used in subsequent analyses. Genes were considered as expressed if TPM > 1 for CAGE data and FPKM > 1 for RNA-Seq data.

### Correlation analysis

Pairwise Spearman correlation coefficients were calculated using FPKM and TPM values of 17,765 genes, which includes all protein-coding genes that show expression in at least one sample in both CAGE and RNA-Seq data sets, for all 27 FANTOM5 samples against all 79 samples from HPA. Both the all-against-all dotplot and heatmap were generated using these correlation values. Hierarchical clustering of FANTOM5 samples was produced using (1 – correlation coefficient) as distance. The average linkage method was used to measure the distance between clusters.

### Categorization of ubiquitously expressed and single-tissue enriched genes

A gene was considered as ‘ubiquitously expressed’ or ‘expressed in all’ if its expression levels were > 1 TPM / FPKM in all tissue samples. Genes that were expressed in 100% of the samples in one data set and in 95% of the other data set (27/27 of FANTOM5 samples and ≥ 75/79 of HPA samples, or 79/79 of HPA samples and ≥ 25/27 of FANTOM5 samples) were considered as ‘in agreement’ by both data sets. A gene was considered as single tissue-enriched if the gene expression was at least *x*-fold higher in one particular tissue than all other tissues in that data set. More relaxed criteria were also tested, where genes were counted as ‘in agreement’ for the single tissue-enriched category if the tissue with the highest expression for that gene was the same for both data sets and if the gene expression had a >*x-*fold enrichment for one data set and >*y-*fold higher for the other data set (where *y* < *x*). Fold enrichment values tested for *x* included 3-, 5-, 7-, and 10-fold, and for *y*, 3- and 5-fold. If a gene belonged to both ubiquitously expressed and single tissue-enriched categories, it was counted as a single tissue-enriched gene for visualization purposes in the scatterplots (fewer genes belong to this category). Statistical significance of proportions of genes in specified categories were calculated using Student's *t*-test.

### Gene model comparison

For genes that belonged to the HPA-only or FANTOM5-only ubiquitously expressed or single tissue-enriched categories, manual examination was performed on the most extreme cases (expressed in all in one data set and expressed in 0 or in a few samples in the other data set). For cases where expression was low or zero in FANTOM5 samples, a search for CAGE peaks with genomic locations greater than 500 bp upstream and downstream from the gene's known start sites was performed. Identified CAGE peak(s) were checked for tissue specificity and expression levels using Zenbu genome browser to see how well the peak(s) compared to that of the HPA data set (21). For dubious RNA-Seq read coverage plots, the sequence of the stretch of mapped reads was checked against the transcriptome to see if it mapped to other genes.

### Evaluation of CAGE peak distance to annotated transcript start sites (TSS)

To see how genes with RNA-Seq expression map to CAGE peaks >500 bp from the annotated TSSes, firstly, for all 20,940 protein coding genes, the distance of the closest CAGE peaks was identified by the closest TSS for each gene. Secondly, the 184,827 robust CAGE peaks from FANTOM5 data were mapped to the closest annotated TSSes for all genes. The subset of genes with RNA-Seq expression but without CAGE expression in brain, pancreas, placenta, and testis were then used to create scatterplots showing RNA-Seq expression versus distance of each gene's annotated TSSes to the closest corresponding CAGE peaks for each tissue.

## RESULTS

### Data set overview

We compared both coding and non-coding genes from 22 corresponding tissues, with 79 samples from the HPA RNA-Seq data set and 27 samples from the FANTOM5 CAGE data set. The overall number of protein coding genes expressed in each tissue ranges from 12,135 genes in liver to 15,509 genes in testis for the HPA data set and 12,423 in heart tissue to 14,588 genes in testis in the FANTOM5 data set. Between corresponding tissues, the percentages of agreement, in terms of which genes are expressed, range from 84% to 88% (Supplementary Table S1). The number of non-coding genes (ncRNAs) ranges from 2163 to 6650 genes in the HPA RNA-Seq data set and 1380 to 2607 genes in the FANTOM5 CAGE data set, with about 22–25% agreement for each corresponding tissue. Salivary gland, liver and pancreas samples exhibit genes with the overall largest expression ranges for coding genes while ovary and uterus had the most narrow expression ranges in both data sets (Supplementary Figure S1). Due to the low percentages of agreement for non-coding genes, we decided to focus the remainder of this study on comparisons of protein-coding genes only.

### Overall gene expression is comparable between FANTOM5 and HPA data sets

**Figure 2.**
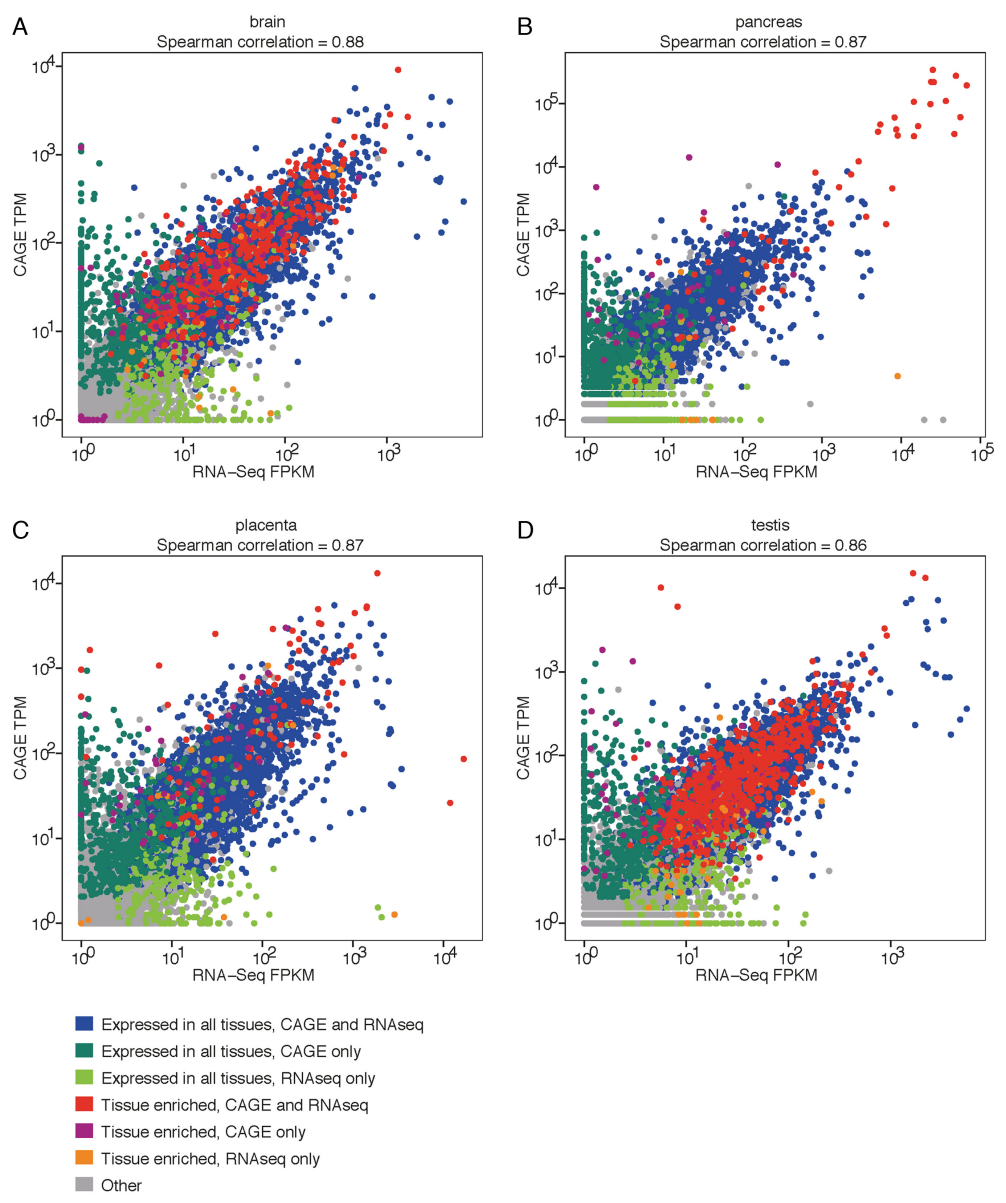
Gene expression correlations between corresponding tissues in the FANTOM5 CAGE and HPA RNA-Seq data sets. Scatterplots of gene expressions measured in TPMs for CAGE data set and FPKM values for RNA-Seq data set are shown for (**A**) brain, (**B**) pancreas, (**C**) placenta and (**D**) testis. The axes are shown in log10 scales. Only protein-coding genes mapped in both data sets are used in this analysis.

Gene expression of corresponding tissue samples from the FANTOM5 and HPA data sets were used for tissue-against-tissue comparison. Four representative tissue comparison plots are shown in Figure 2 (comparisons for the other 18 tissues can be found in Supplementary Figure S2). To facilitate with the interpretation of the scatterplots, we categorized the genes into ‘Expressed in all’, defined as genes expressed in all tissues, ‘Single tissue-enriched’, shown here as at least 7-fold higher in one particular tissue compared to all other tissues, and ‘Other’, which are genes that belong to neither categories. Expression levels of ubiquitously expressed genes common to both data sets (defined as expressed in all tissues in one data set and found in 95% of the tissues in the other data set) tend to have higher expressions than ubiquitously expressed genes found in one data set only. Single tissue-enriched genes are mostly found in brain and testis by far, confirming previous observations (17). The high global correlations for all tissue comparisons suggest that overall, the same genes were detected/expressed at similar levels in both data sets for each tissue (Figure 2, Supplementary Figure S2). This was confirmed by examining gene expression across 22 tissues in terms of maximum expression values over mean values (Supplementary Figure S3). Higher max/mean values suggest higher specificity of gene expression in few tissues. Of the max/mean ratio differences, 95% of genes are within ±6 between the two data sets. We also looked at max/median value comparison between FANTOM5 and HPA, and we found that ∼94% of the gene expression ratio differences are within 10-fold. Besides salivary gland, where the expression levels of the top expressed genes were one order of magnitude higher in the FANTOM5 data set, the magnitudes of gene expression seemed comparable for protein-coding genes in most tissues.

### Tissue expression signatures are independent of the data set and profiling method

**Figure 3.**
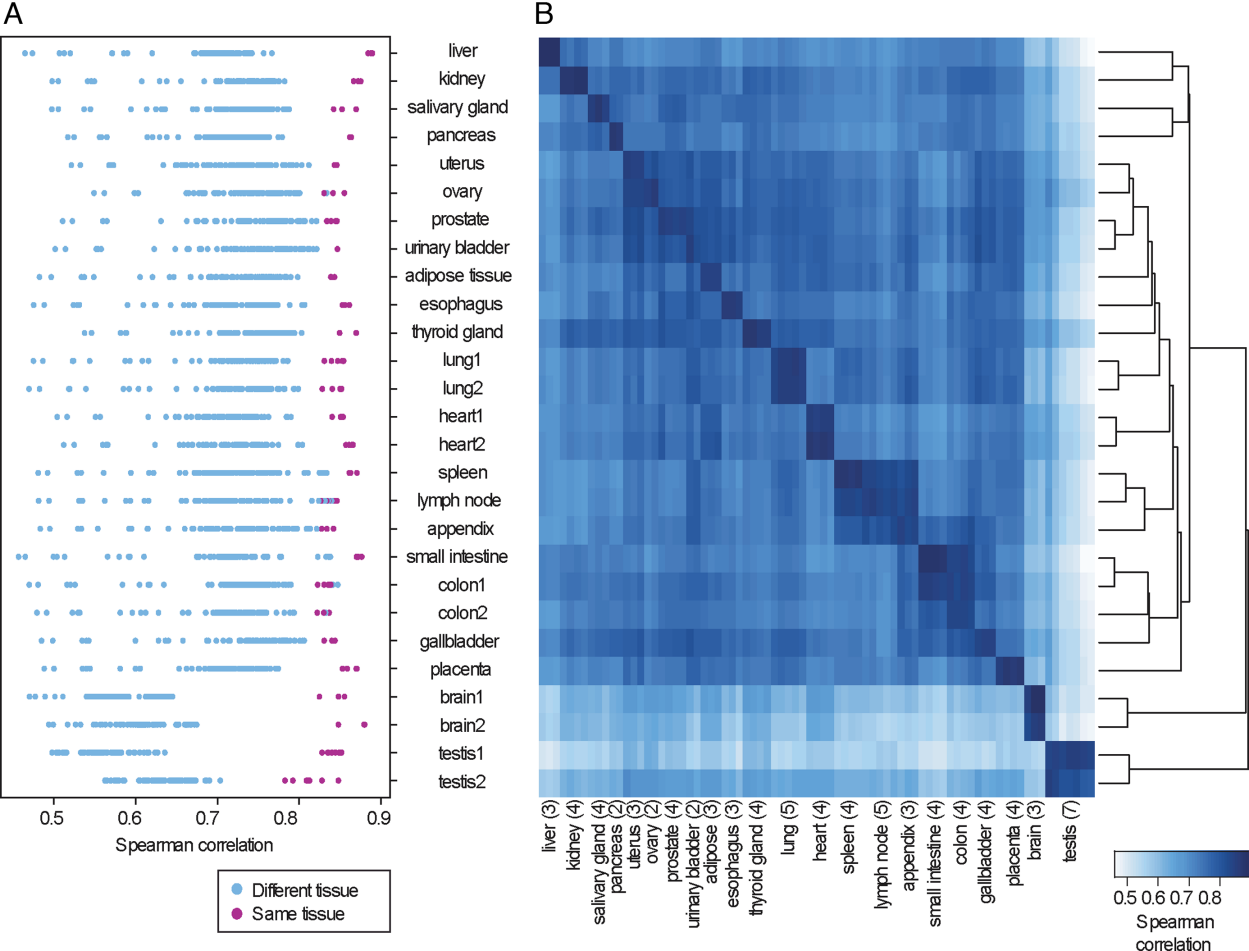
Comparison of overall correlation values between 22 tissue samples chosen from the FANTOM5 and HPA data sets. (**A**) The dotplot shows the ranges of correlation values between each of the 27 tissue samples in FANTOM5 data set against all of the 75 HPA tissue samples (brain, colon, heart, lung, and testis each has two samples coming from the same tissue). (**B**) Hierarchical clustering shows tissue relationships within the 27 FANTOM5 samples. The heatmap shows subtle differences in the correlation relationship of HPA tissue samples to FANTOM5 tissues samples. All correlation scores were calculated as pair-wise Spearman correlation coefficients between the tissue samples.

Because the ubiquitously expressed genes accounted for 50–70% of all expressed genes within each tissue, we wanted to know if overall gene expression distinguishes one particular tissue from all other tissues. We found that for 18 out of 22 of the FANTOM5 tissues, Spearman correlation values showed clear distinction between corresponding tissues versus other tissues (*R* > 0.8) (Figure 3A). For prostate, ovary, lymph node, appendix, and one of the CAGE colon samples, the correlation values between corresponding tissues were not so well separated from the next most closely related tissues. Correlation coefficients for non-corresponding tissues between the two data sets mostly ranged from 0.65 to 0.8. Brain and testis had overall lower correlations to other tissues. To check if the high number of testis- and brain-specific genes were solely responsible for the low correlation levels, we tried computing correlation levels with all genes expressed in both data set minus all the CAGE and RNA-Seq testis- and brain-enriched genes. We found the correlation range for one testis tissue became much more similar to the other tissues (Supplementary Figure S4). However, for both FANTOM5 brain samples and one of the testis samples, the correlation ranges remained lower than for other tissues, in the range of 0.55 to 0.7.

Tissue groupings were in agreement between HPA and FANTOM5 samples, with subtle differences between the two data sets, as shown by asymmetric color shading of the heatmap in Figure 3B. Major groupings include immune system organs (spleen, lymph node, appendix), digestive system (appendix, colon, small intestine), and organs consisting of similar cell types (uterus, ovary, prostate, urinary bladder). Overall, the subtle shading asymmetry may reflect the subtle tissue composition differences, yet the strong correlations between the corresponding tissues from the two data sets confirm the distinction of tissue expression signatures, especially for distinct organs.

### Global comparison of ubiquitously expressed and single tissue-enriched genes demonstrate data set comparability

**Figure 4.**
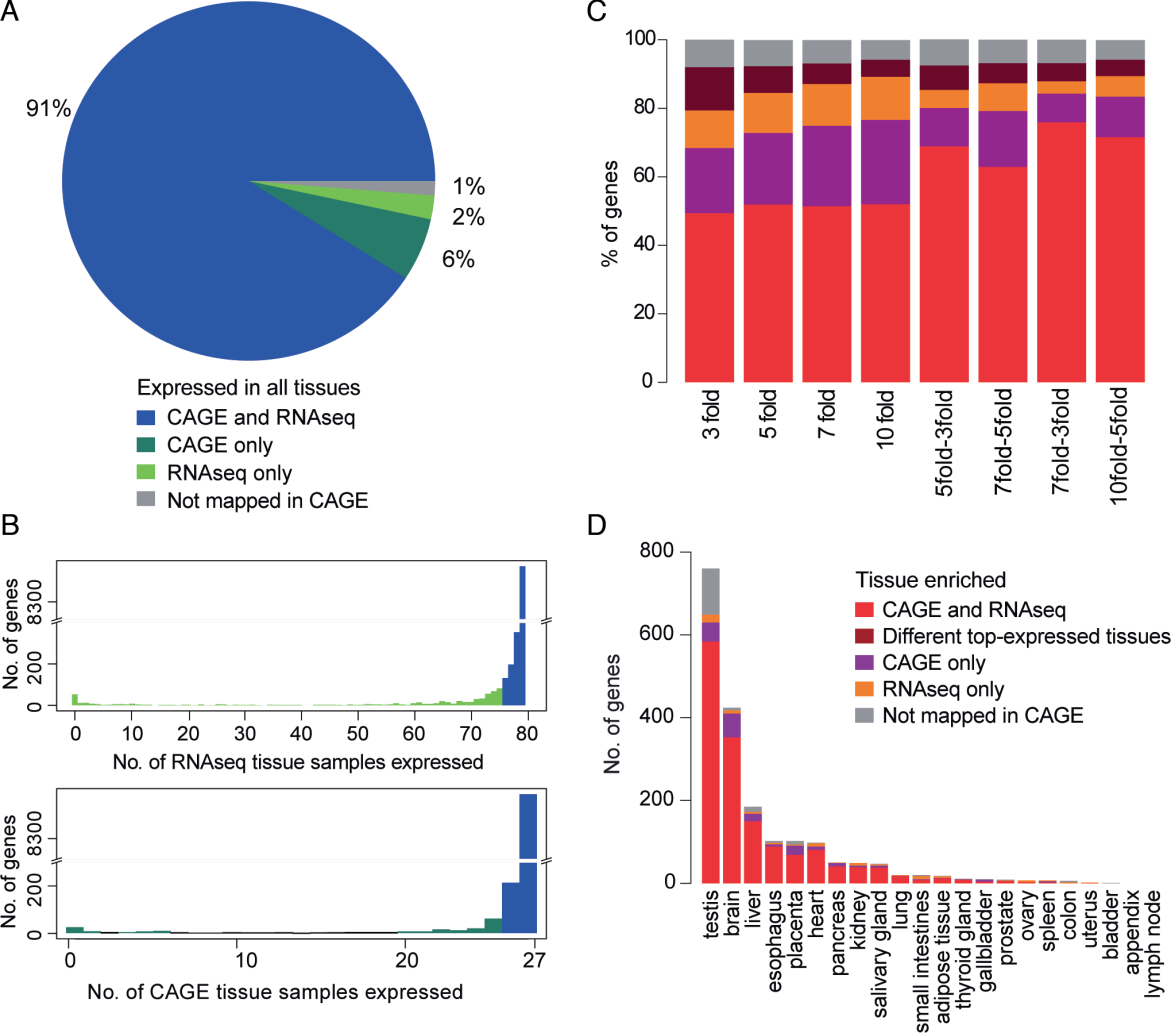
Distribution of ubiquitously expressed and single tissue-enriched genes among FANTOM5 CAGE and HPA RNA-Seq data sets. (**A**) Distribution of ubiquitously expressed genes in either FANTOM5 or HPA data set. A gene is considered as ubiquitously expressed for both data sets if it is expressed in all samples in one data set and in 95% of the tissues in the other data set. (**B**) Top histogram shows distribution of FANTOM5 ubiquitously expressed genes in HPA RNA-Seq tissue samples. Bottom histogram shows distribution of HPA ubiquitous gene expression in FANTOM5 CAGE tissue samples. The histograms confirm that most of the genes are expressed in 95% of the tissues in the other data set. (**C**) Distribution of single tissue-enriched genes among 22 tissues identified in either FANTOM5 or HPA data set for 3-, 5-, 7-, 10-fold, 5-fold–3-fold, 7-fold–5-fold, 7-fold–3-fold, and 10-fold–5-fold enrichment. (**D**) Tissue distribution of the single tissue-enriched genes identified in FANTOM5 and HPA tissue samples, shown for 7-fold–3-fold enrichment.

To assess how gene expressions compared at a global level, we took a closer look at the level of agreement between the two data sets for two groups of genes: genes that were ubiquitously expressed in most tissues and genes that were highly tissue-specific or highly enriched in one tissue, to see how well the two data sets coincide. We found that 91% of the ubiquitously expressed gene category were common to HPA RNA-Seq and FANTOM5 CAGE data (Figure 4A). A histogram is shown for RNA-Seq ubiquitous genes’ expression in CAGE, and vice versa, to show that indeed ∼50% of the data set specific ubiquitous genes were expressed in 95% of the tissues in the other data set (Figure 4B). 8% of the CAGE-only ubiquitous genes and 7% of the RNA-Seq-only ubiquitous genes were found to be absent in the other data set, suggesting some sort of technical detection issues, which will be discussed further in the next section. For single tissue-enriched genes, different fold enrichments show that the agreements between two data sets were around 50% for strict fold comparisons. The agreements increased to 63–75% when more relaxed fold enrichment criteria were employed (Figure 4C). We further examined the single-tissue enriched genes by their distributions among different tissues, and found that for all enrichment cut-offs examined, the majority of the single tissue-enriched genes were expressed in testis (38%, *P* < 3 × 10^−10^), followed by brain (21%, *P* < 3 × 10^−9^) and liver (10%, *P* < 3 × 10^−5^) (7-fold–3-fold comparison shown as an example in Figure 4D; comparisons for other cutoffs are shown in Supplementary Figure S5). The order of the other tissues were less consistent, though immune tissues and urinary bladder were consistently found to have the lowest numbers of single tissue-enriched genes (bladder enriched genes = 0.1%, *P* < 1 × 10^−12^; lymph node enriched = 0.2%, *P* < 1 × 10^−10^). Looking at strict cutoffs (3-, 5-, 7-,10-fold enrichment) versus relaxed cutoffs (5-fold–3-fold, 7-fold–3-fold, 7-fold–5-fold, 10-fold–5-fold), we found the proportion of CAGE-only brain-enriched genes to be higher for strict cutoffs (9% versus 4%, *P* < 5 × 10^−3^), whereas for testis-enriched genes, the proportion of RNA-Seq- only enriched genes were significantly higher using strict cutoffs (5% versus 2%, *P* < 4 × 10^−3^). Overall, CAGE and RNA-Seq data sets largely agreed in terms of ubiquitously expressed genes, whereas single-tissue enriched genes showed higher agreements with more relaxed fold enrichment cutoffs. The lists of genes in each category of commonly and single data set only ubiquitous genes and single-tissue expressed genes for 7-fold–3-fold comparison can be found in Supplementary Tables S2 and S3.

### Discrepancies between two data sets can largely be explained by gene model annotation issues or by observing immunohistochemistry-based protein profiles

We identified seven factors which could explain the discrepancies between the two data sets, and we attempt, as much as we could, to find out what proportion of the discrepancies can be explained by each of these factors (Table 1).

**Table 1.** List of potential reasons for discrepancies between FANTOM5 CAGE and HPA RNA-Seq data sets

Type of Discrepancy	Reason	Approximate proportion of cases affected
Data set 1 expression low; Data set 2 expression 0	Detection limit (different sequencing depths between data sets)	60% of CAGE-only and 67% of RNAseq-only ubiquitously expressed genes
Data set 1 expression low; Data set 2 expression 0	Read-throughs, anti-sense RNAs, unconfirmed genes (annotations updated in new version of Ensembl annotation)	Up to 90% of the FANTOM-only ubiquitous genes with 0 expression in RNA-Seq
CAGE expression high; RNA-Seq expression low or 0	PolyA(-) mRNAs not measured by RNA-Seq	∼22% of CAGE only ubiquitous genes
RNA-Seq expression positive; CAGE expression 0	CAGE peaks outside of 500 bp range	∼1% of RNA-Seq expressed genes have CAGE peaks 500–1500 bp from annotated TSS
RNA-Seq expression positive; CAGE expression 0	Multi-mapping CAGE peaks not included in data set	up to 9% of human proteome (∼1800 genes) not supported by robust CAGE peaks
Data set 1 expression high; Data set 2 expression low	Difference in specific cell type proportions between corresponding tissues	Hard to estimate; no premium antibody staining for all annotated genes
CAGE expression in a single tissue; RNA-Seq expression in multiple tissues	CAGE peak maps to one gene; RNA-Seq reads map to multiple genes	< 1% of discrepancy genes, as most highly homologous genes were not mapped in CAGE data (Example in Supplementary Figure S8c)

To investigate in discrepancies caused by detection limit, where tissue samples in one data set may have had deeper sequence depth or stricter filtering criteria than the other data set, we looked into the 8% of disagreeing ubiquitous gene cases. The genes found to not meet the strict criteria tended to have lower expressions overall, with 60**%** of CAGE-only ubiquitous genes and 67% of RNA-Seq only ubiquitous genes having 1 or more samples with expression < 3 TPMs/FPKMs, which could explain why they were undetected in the other data set. In addition, within the 40 FANOMT5-only ubiquitous genes with no expression in the RNA-Seq data, 80% of them were uncharacterized proteins, read throughs, or have no annotations at all (Supplementary Table S3).

For cases where CAGE expression was high and RNA-Seq had little or no expression, a main reason is that polyA(-) genes are not detected by the standard RNA-Seq sample preparation protocol. Given that histone genes are the only group of well-known polyA(-) genes, we took FANTOM5-only ubiquitous genes and compared the gene list with polyA(-) and bimorphic genes (defined as genes occurring in both poly(A)+ and poly(A)- forms) from Yang *et al.’*s study of non-polyadenylated RNA (22). We found that that ∼22% of the genes in this group overlap with the polyA(-) and bimorphic gene lists for HeLa and H9 cells (including 34 histone genes). From this we deduce that at least 20% of the genes expressed only (or with much higher expression) in the FANTOM5 data set may be lowly or selectively poly-adenylated and are thus not fully captured in the HPA RNA-Seq data set.

For cases where RNA-Seq showed expression and CAGE data showed no expression, known reasons include 1) CAGE peaks mapping >500 bp from annotated TSS, and 2) CAGE peaks mapping to multiple locations on the human genomes were removed from the FANTOM5 data set to reduce false reports of expressions (15). To estimate how many TSSes were potentially located outside of ±500 bp window of annotated TSSes, we identified the distance to the closest CAGE peaks for each annotated protein coding genes (Supplementary Figure S6) and found 1,270 genes with CAGE peaks located >500 bp from annotated TSSes. We then looked at the distance of the closest CAGE peak for all genes with RNA-Seq expression in at least one of the tissue samples. We found that 184 genes (∼1% of RNA-Seq expressed genes) were between 500 and 1500 bp, and another 165 genes had closest CAGE peaks mapped between 1500 and 10 000 bp of annotated TSSes. By examining the higher RNA-Seq expressed genes in brain, pancreas, placenta, and testis, we found that CAGE peaks up to 1500 bp away from annotated TSS may still correspond to RNA-Seq expression (Supplementary Figure S7). The FANTOM5 paper noted that the robust CAGE peaks map to 91% of the annotated genes, leaving 9% as not mapped due to either the multi-mapping issue or the TSS >500 bp issue (15).

**Figure 5.**
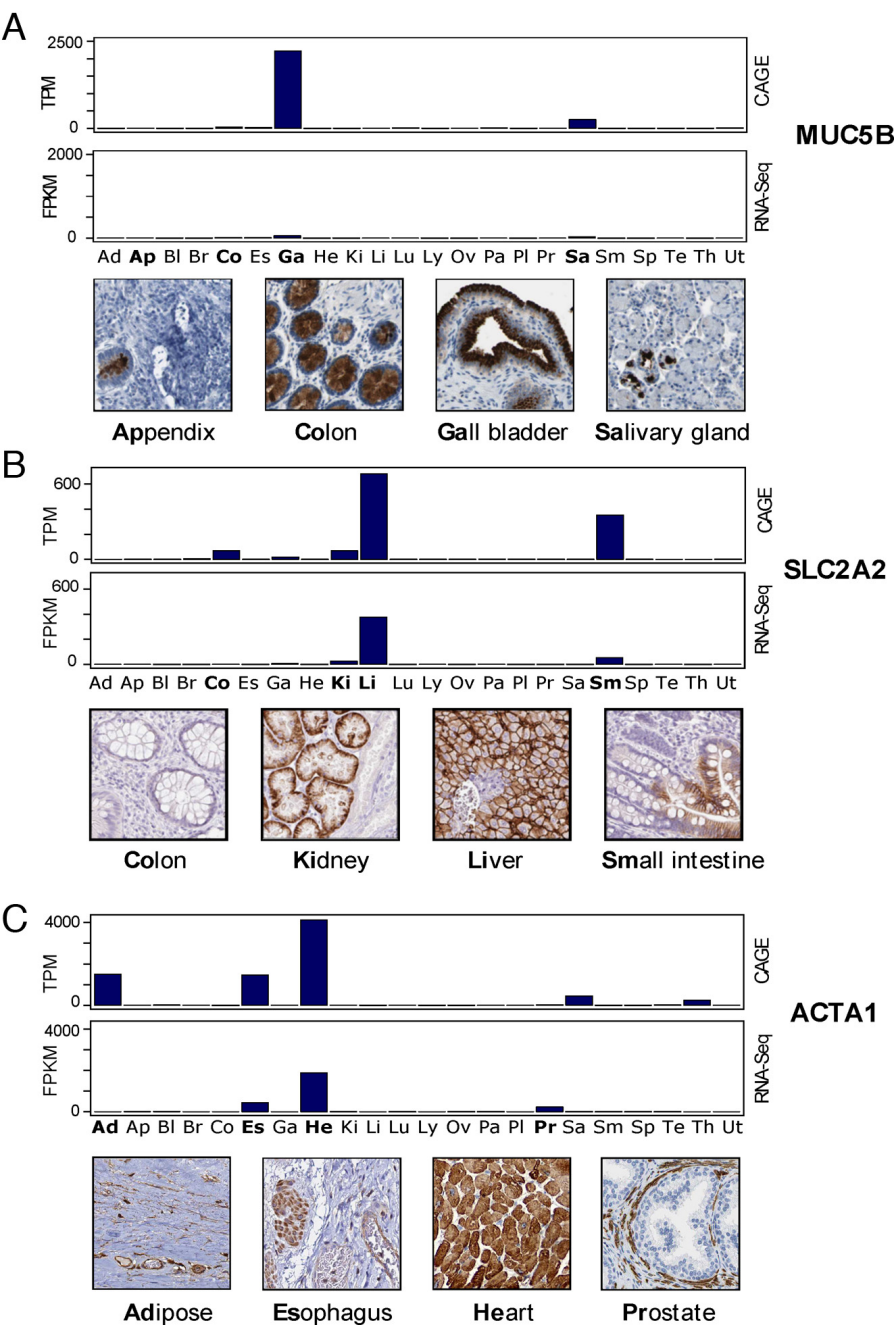
Examples of how HPA immunohistochemistry (IHC) staining can complement gene expression data by providing additional spatial distribution information at the single-cell level. (**A**) MUC5B, a CAGE-only gall bladder-enriched gene in the IHC images showed very distinct cell type-specific expression in gall bladder, which could explain the difference in expression value between CAGE and RNA-Seq. It show high expression in specific cell types in the colon, salivary gland, and even in the appendix, where both CAGE and RNA-Seq expressions were low. (**B**) SLC2A2, an RNA-Seq only liver-enriched gene in the IHC images display highly specific expression in kidney, liver and small intestine, which is in agreement with CAGE data. Colon IHC image shows no expression, suggesting that one of the FANTOM5 colon samples may be contaminated. (**C**) Variable composition of certain cell types: ACTA1 shows tissue-restricted expression in adipose, esophagus, heart, salivary gland, and thyroid in CAGE. RNA-Seq data does not show high expression in adipose tissue, salivary gland or thyroid. Examination of IHC images shows very specific staining patterns for muscle cells in each tissue sample, which explains the variable expressions of this gene between the two data sets. Tissue abbreviations are as following: Ad = adipose, Ap = appendix, Bl = bladder, Br = brain, Co = colon, Es = esophagus, Ga = gallbladder, He = heart, Ki = kidney, Li = liver, Lu = lung, Ly = lymph node, Ov = ovary, Pa = pancreas, Pl = placenta, Pr = prostate, Sa = salivary gland, Sm = small intestine, Sp = spleen, Te = testis, Th = thyroid, Ut = uterus.

Reasons for genes having high expression in one data set and low expression in the other data set are more complex. We looked at the 17% of genes that were in ‘disagreement’ in the 7-fold–3-fold single-tissue enriched category. Since tissue cell type proportion difference could not easily be determined using sequencing data alone, we used HPA's antibody-based immunohistochemistry (IHC) protein profiling images as a validation source. Using only genes overlapping with ‘premium’ antibody profiling, we identified the following examples. Figure 5A shows MUC5B, a gene identified to be CAGE-only gall bladder-enriched gene. In RNA-Seq, the expression of MUC5B in gall bladder was not much higher than in colon and small intestine. IHC images show that MUC5B is actually expressed in selected mucous producing cells and glandular cells in gall bladder, colon, and highly specifically within a small proportion of cells in the salivary gland. IHC also shows specific expression in appendix that was not picked up by either data set. Depending on the proportion of different cell types within each tissue sample, the expression values of this gene can vary a lot, which could explain the discrepancy between CAGE and RNA-Seq results. Figure 5B shows SLC2A2, an RNA-Seq-only liver-enriched gene. However, in CAGE data it is also highly expressed in small intestine, with some expression in colon, gall bladder, and kidney. The IHC images confirm expression in liver, small intestine, and kidney, but not for colon. One of the colon samples in CAGE had been identified to have overall gene expression more similar to RNA-Seq small intestines than to RNA-Seq colon, suggesting possible contamination (Figure 3B). This could explain why the IHC colon staining showed disagreement with CAGE results. In the third example (Figure 5C), ACTA1, a skeletal muscle alpha actin gene, which showed some enrichment in adipose tissues in the CAGE data set but not for the RNA-Seq data set. The IHC images show that all tissues that contain some muscle cells showed positive staining, implying that the FANTOM5 adipose sample contained a higher proportion of muscle cells than HPA adipose samples. These examples illustrate that ‘disagreement’ between the gene expression data may simply have been natural variations that occur within tissues or that expression is localized to a small proportion of specific cells that may not be included in all samples of that tissue type, and may have been resolved if there were more tissue replicates for both FANTOM5 and HPA data sets.

## DISCUSSION

We have shown that despite large differences in tissue preparation, RNA processing procedures, sequencing technologies, and computational mapping/quantification methods, the two tissue transcriptome data sets are largely comparable at the gene level. This has several implications. First, it shows that transcriptome profiling of healthy tissues is quite comparable even with different processing methods, if high quality experimental and computational pipelines were employed. It also suggests that these two data sets could be used to complement each other as reference transcriptome profiles. In addition to providing full exon/transcript information, the HPA RNA-Seq data set has the advantages of having high quality replicates for all tissues, supplemented by clinical information on each sample (such as gender, age, estimate of percentage of major cell types), as well as protein IHC data. On the other hand, the FANTOM5 CAGE data set provides breadth for more specialized tissues such as specific eye muscles and many more brain regions, as well as primary cell data. In terms of genome coverage, FANTOM5 chose strict mapping criteria where any of the CAGE reads that mapped to multiple locations in the genome were discarded. The RNA-Seq data set followed Cufflinks’ mapping strategy by splitting the reads between all the locations mapped, which reduced quantitation accuracy for some of the genes, but allowed more reads to be mapped. This means that RNA-Seq can provide better presence/absence information for genes not mapped by CAGE, but CAGE can potentially help identify false positive gene expression in RNA-Seq, as shown by the examples in Supplementary Figure S8. The FANTOM5 data set provides better quantification data for histones and other genes for which mRNAs are poly(A)- or bimorphic. Ideally, the integration or at least direct linkage of the two data sets would provide synergistic results of refining gene models, validating quantitation levels, and/or showing broader ranges of expression variation for each gene. With eventual improvements in gene annotation and advancements in read mapping/transcript quantification algorithms, there is potential to further increase the comparability between the FANTOM5 and HPA data sets just by reprocessing the two raw data sets with improved computational pipelines.

We chose to compare the two data sets at the gene level rather than at the transcript level for the reason that RNA-Seq transcript identification and quantitation is still not very accurate with currently available RNA-sequencing protocols and algorithms (23,24). While correlations among CAGE tissue transcriptomes were comparable between the TSS level and the gene level, the correlations between the RNA-Seq tissue replicates at the transcript level were much lower than at the gene level. Given that a significant number of novel transcripts and TSSes have been identified by these two data sets, and given that transcript quantification depends on transcript model annotation, we feel that such comparison will be much more informative once the transcript annotations have been updated accordingly.

We focused most of our analyses on the protein-coding genes, since the level of agreement of non-coding RNA expression between the two data sets was quite low. This is likely due to the fact that 1) exact start sites of ncRNA are likely less well annotated compared to protein coding genes, and 2) the RNA-Seq protocol used for the data set does not efficiently capture polyA(-) RNA, and is not optimized to distinguish anti-sense from sense RNA. For example, MALAT1 has been shown to be the highest expressed gene in most of the CAGE samples (15). However, in RNA-Seq samples it only showed moderate levels of expression due to the majority of the transcripts being polyA(-) (25). It is possible that many of the non-coding genes have different splicing and poly-adenylation patterns compared to coding genes. The availability of both CAGE and RNA-Seq transcriptome data will aid in future research on non-coding RNAs in identifying their structures and tissue-specificities. In terms of coding genes, the next step would be to combine mRNA data to see how they compare with protein quantitation data. Recently, a published study of the draft human proteome found that the protein-to-mRNA ratio is highly correlated with protein abundance levels (26). It has been suggested that mRNA levels determine the approximate magnitude of protein abundance, and post-transcriptional control fine tunes the final protein levels in cells (27). Further questions remain if genes that have consistently high or low protein-to mRNA ratio groups have strong functional associations. The ultimate goal will be accurate prediction of protein abundance directly from RNA quantification.

We used ubiquitously expressed genes and tissue-specific genes as test cases for probing the comparability and discrepancies of the two data sets because the former category tests for consistency of gene expression detection across different samples, and the latter tests for gene expression detection specificity in single tissues samples. Many previous studies have looked into criteria for identifying housekeeping genes among human tissues using microarray, NGS sequencing and proteomics data, and explored their functions, subcellular localizations, as well as other properties that may suggest housekeeping roles (28–31). The combination of FANTOM5 CAGE, HPA RNA-Seq, and HPA IHC protein profiles in tissues is ideal for refining the housekeeping gene list. Future studies could look into identifying genes that are expressed in every cell type, versus genes that are expressed in common general cell types found in most tissues. As for tissue-specific gene expression comparison, the analyses revealed that establishing tissue-specificity was not straightforward. Some genes seemed to be cell type markers (such as ACTA1 for muscle cells), while other genes seemed to be expressed in several different cell types and in a quite specific manner that could only be elucidated by examining the IHC tissue images (such as MUC5B). The twenty-two tissues used in this study were not quite comprehensive enough to cover the entire human body. For example, if we included the bone marrow, skeletal muscles, skin, and perhaps oocytes and sperm, some of the single tissue-enriched genes from the current study might also be expressed in these tissues. In addition, more specific organs such as the eye or the inner ear would likely express certain interesting organ-specific genes not identified in this paper. We hypothesize that many of the ‘testis-specific’ genes, especially the ones related to meiosis and gametogenesis, would also be expressed in oocytes and in fetal ovary undergoing oogenesis (32). Some efforts have been put into identifying tissue-specific genes either with global studies or looking at individual tissues (32–37). Further efforts could focus on developing more sophisticated measures to establish better criteria for identifying cell type proportions, robust tissue signatures, or tissue state markers. Such study would make use of all the 149 tissues available from the FANTOM5 data, as well as other RNA-Seq human tissue data sources such as GTEx (11) and Expression Atlas (12).

We identified factors explaining discrepancies between the two data sets, which include gene annotation errors, read mapping issues, cell type proportions, detection limit, and polyA(-) genes under-detected by RNA-Seq polyA(+) enrichment protocol (38). The fact that most FANTOM5 samples did not have replicates probably also contributed to the variations and discrepancies in the comparisons. The IHC results demonstrate that many genes identified as enriched in certain tissues were found to be concentrated in specific cell types among other tissues, with diverse cell type composition variations. Furthermore, some of the genes that differ significantly between the two data sets are actually markers for contaminating cell types. For example, higher values of ACTA1, and MYH7 (myosin) in FANTOM5 adipose tissue indicate muscle cell contamination, whereas higher values of HBA1 and HBA2 indicate blood cell contamination in HPA adipose tissue. Other marker genes could potentially be used to estimate the percentage of certain cell types in any transcriptome profiling of tissues. In terms of detection inconsistency of lowly expressed genes, using the permissive FANTOM5 promoter data set to identify gene expression may improve comparability (15). In addition, factors such as RNA length, GC bias, normalization methods, sequence biases specific to each technology, along with the biases and limitations within the computational pipelines used to process the sequencing data also attribute to the discrepancies. The current HPA contains some antibodies with non-specific off target binding (17), but by restricting analyses to antibodies with higher reliability scores and with improvements made to the protein atlas, the IHC could provide useful validation for transcriptome data for most of these discrepancy cases. Conversely, the consensus results of CAGE and RNA-Seq data could be used to aid with validating antibody specificity and improve the IHC data generation process.

We have shown that the integration of CAGE, RNA-Seq and HPA IHC data can help refine gene models and improve the interpretation of gene expression values. In the future, integration of other types of high quality high-throughput gene expression data would further refine the transcription/translational profiles of the human body. The GTEx project (11), with tissue transcriptomes from many normal individuals, would provide data on frequencies as well as normal expression ranges for all human tissue-expressed genes, along with common mutations carried within individuals. In addition, the two recently published mass-spectrometry-based human proteomes can of course also serve as valuable references (26,39). Integrating other, more tissue-specific transcriptome data sets, such as BrainSpan (http://www.brainspan.org), could help us improve the identification and definition of tissue-specific genes. Proteomics data measured by alternative technologies are especially useful for validation of HPA's antibody-based protein profiling for cases when mRNA levels of a gene do not correlate well with protein expression levels, as well as providing expression evidence for proteins and peptides from non-coding RNAs (27,40,41). By extending the comparison to other organisms, such as the Non-human Primate Reference Transcriptome Resources (42), we can further understand conservation of gene expression within different tissues from an evolutionary aspect. With the availability of more high quality transcriptomics and proteomics data resources generated using different technologies, effective efforts of data integration has excellent potential for aiding the discovery of underlying mechanisms for complex diseases, novel disease diagnosis methods, as well as the development of novel treatments.

## SUPPLEMENTARY DATA

Supplementary Data are available at NAR Online.

SUPPLEMENTARY DATA
