# Supplementary material for: Complementing tissue characterization by integrating transcriptome profiling from the Human Protein Atlas and from the FANTOM5 consortium
Source: Nucleic Acids Res. 2015 Jun 27;43(14):6787–98. doi: 10.1093/nar/gkv608 (PMC4538815; doi:10.1093/nar/gkv608)
Supplement: SUPPLEMENTARY DATA [file supp_43_14_6787__index.html]

Complementing tissue characterization by integrating transcriptome profiling from the Human Protein Atlas and from the FANTOM5 consortium — SUPPLEMENTARY DATA 

# Complementing tissue characterization by integrating transcriptome profiling from the Human Protein Atlas and from the FANTOM5 consortium

## SUPPLEMENTARY DATA

- SUPPLEMENTARY DATA
- SUPPLEMENTARY DATA
- SUPPLEMENTARY DATA
- SUPPLEMENTARY DATA
- SUPPLEMENTARY DATA
