# Supplementary material for: Complementing tissue characterization by integrating transcriptome profiling from the Human Protein Atlas and from the FANTOM5 consortium
Source: Nucleic Acids Res. 2015 Jun 27;43(14):6787–98. doi: 10.1093/nar/gkv608 (PMC4538815; doi:10.1093/nar/gkv608)
Supplement: SUPPLEMENTARY DATA [file supp_gkv608_nar-00656-h-2015-File007.doc]

# Legends for Supplementary Data

# SupplementaryFiguresS1-S8.pdf Additional_file_2.xls Additional_file_3.xlsx Additional_file_4.xls

### Supplementary Figure S1. Boxplots for overall gene expression distributions.

a) Protein-coding gene expression distributions for FANTOM5 CAGE tissue samples. b) Protein-coding gene expression distributions for HPA RNA-Seq samples. c) Non-coding gene expression distributions for FANTOM5 CAGE tissue samples. d) Non-coding gene expression distributions for HPA RNA-Seq samples.

### Supplementary Figure S2. FANTOM5 vs. HPA gene expression scatterplots for the other 18 tissues.

Gene expression scatter plots for the other 18 corresponding tissues between FANTOM5 CAGE and HPA RNA-Seq samples not shown in main Figure 2 (one representative replicates chosen for each tissue).

**Supplmentary Figure S3. FANTOM5 vs. HPA max/mean and max/median for all protein-coding gene expressions.**

a) Scatterplort for max over mean gene expressions and b) max over median gene expressions. The color coding scheme are the same as in Figure 2 and Supplementary Figure S2.

**Supplmentary Figure S4. Correlation values between 27 FANTOM5 tissue samples with 75 HPA samples using common genes expressed in both datasets minus brain and testis-enriched genes.**

Correlation values are calculated using genes expressed in both datasets minus brain- and testis-enriched genes identified in either dataset in order to check for brain and testis correlation to other tissues without the bias of tissue-specific genes.

**Supplementary Figure S5. Distribution of single tissue-enriched genes among 22 tissues identified by FANTOM5 CAGE and HPA RNAseq datasets using various fold enrichment cut-offs.**

Strict 3, 5, 7, and 10 fold cut offs are tested, as well as 3fold-5fold, 5fold-7fold, and 10fold-5fold cut-offs to check for distribution of resulting distribution of single-tissue enriched genes.

**Supplmentary Figure S6 .Frequency of CAGE peak distances to annotated TSSes.**

Distribution of closest CAGE peaks to closest annotated TSSes for all annotated protein-coding genes, with a cut-off at ±10,000 bp. The majority of the CAGE peaks cluster near the 5’ start site of annotated TSSes, but the spread of the distance distribution goes beyond 10,000 bp.

**Supplmentary Figure S7. CAGE peak distance to closest annotated TSSes for genes with RNA-Seq expressions.**

CAGE peak distances to the closest protein-coding genes are plotted for a) brain, b) pancreas, c) placenta and d) testis tissue samples with RNA-Seq expressions. Highly expressed genes with corresponding CAGE expression are labeled. Gene names in brackets likely do not correspond to the aligned CAGE peaks since the expression levels do not match between the FANTOM5 and HPA data for those genes.

**Supplmentary Figure S8. Examples of gene models that can be improved by combining CAGE and RNA-Seq expression profiles.**

a) An example where CAGE shows interesting start site pattern for brain expression of CNDP1 and confirms novel transcript start site downstream of known first exon for liver. b) An example of a transcript start site >500bp upstream of all annotated TSSes for KLK13. c) An example of RNA-Seq mapped reads not corresponding to any annotated transcript models for UBBP4. CAGE confirms that the gene is testis-specific rather than expressed in all tissues.

### Additional_file_2.xls - Supplementary Table S1. Sample summary information

Summary information for the FANTOM5 CAGE and HPA RNA-Seq samples, including sample IDs, number of protein-coding and non-coding genes for each sample, number of shared coding and non-coding genes, and Spearman correlation values for corresponding FANTOM5 and HPA tissue samples.

### Additional_file_3.xlsx – Supplementary Table S2. Ubiquitously expressed gene lists.

This table contains the following gene lists: genes that are considered as expressed in 100% of the tissues in both FANTOM5 and HPA data, genes expressed in all samples in one dataset and 95% of the samples in the other dataset, genes expressed in all tissues only in FANTOM5 data, genes expressed in all tissues only in HPA data, genes expressed in all tissues in HPA data and are not mapped in FANTOM5 CAGE dataset, genes expressed in all tissues in FANTOM5 data and no expression in HPA data, and genes expressed in all tissues in HPA data and no expression in FANTOM5 dataset.

### Additional_file_4.xls – Supplementary Table S3. Single tissue-enriched gene lists.

This table contains the following gene lists: genes found to be single-tissue enriched in both FANTOM5 and HPA datasets, single-tissue enriched genes in FANTOM5 dataset only, single-tissue enriched genes in HPA dataset only, single-tissue enriched genes in HPA dataset and not mapped in FANTOM5 CAGE dataset, and genes that are 7-fold enriched in a particular tissue in one dataset but the top expressed tissue differs in the other dataset.
