# Supplementary material for: Complementing tissue characterization by integrating transcriptome profiling from the Human Protein Atlas and from the FANTOM5 consortium
Source: Nucleic Acids Res. 2015 Jun 27;43(14):6787–98. doi: 10.1093/nar/gkv608 (PMC4538815; doi:10.1093/nar/gkv608)
Supplement: SUPPLEMENTARY DATA [file supp_gkv608_nar-00656-h-2015-File008.pdf]

Figure S1

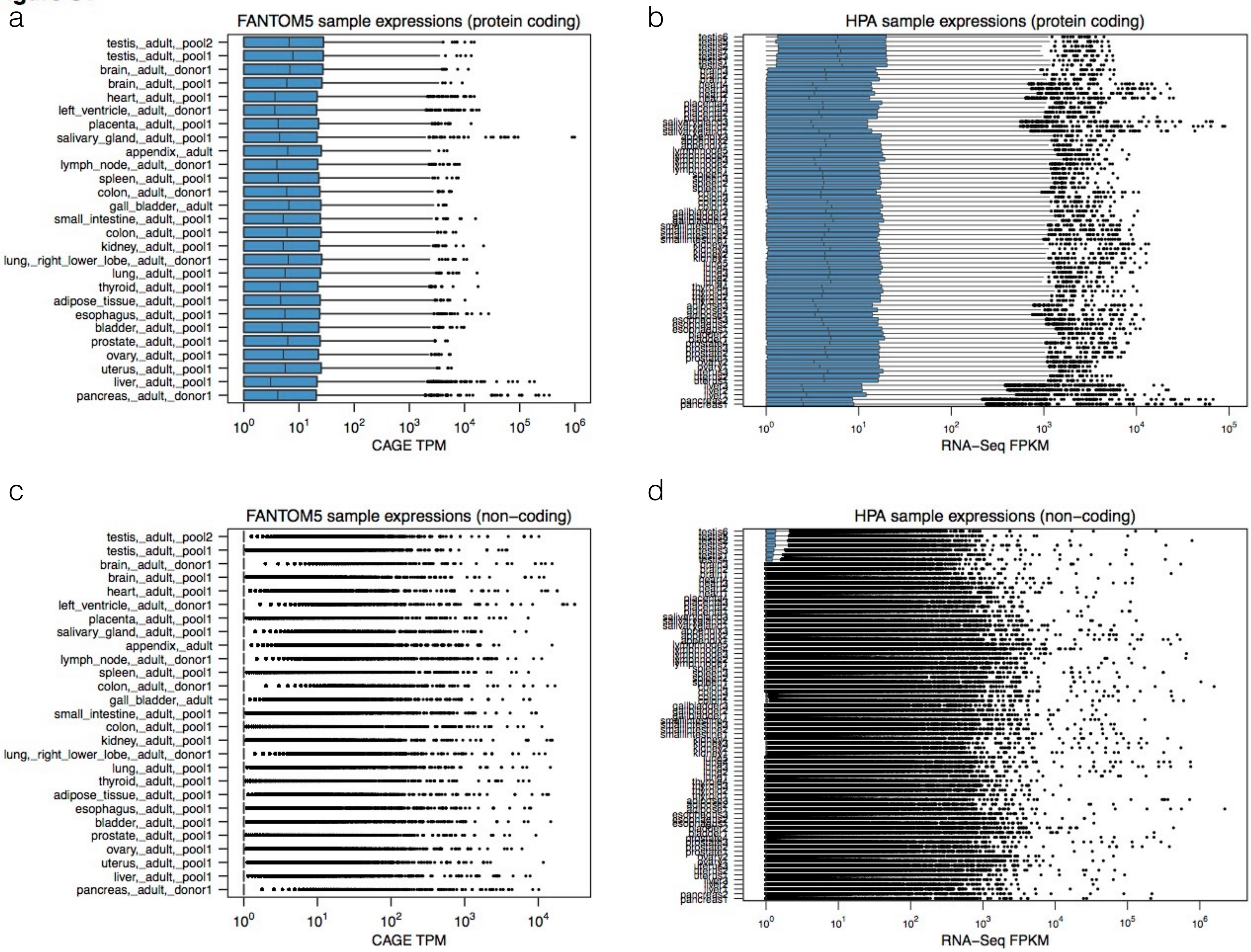
a
FANTOM5 sample expressions (protein coding)
testis,_adult,_pool2
testis,_adult,_pool1
brain,_adult,_donor1
brain,_adult,_pool1
heart,_adult,_pool1
left_ventricle,_adult,_donor1
placenta,_adult,_pool1
salivary_gland,_adult,_pool1
appendix,_adult
lymph_node,_adult,_donor1
spleen,_adult,_pool1
colon,_adult,_donor1
gall_bladder,_adult
small_intestine,_adult,_pool1
colon,_adult,_pool1
kidney,_adult,_pool1
lung,_right_lower_lobe,_adult,_donor1
lung,_adult,_pool1
thyroid,_adult,_pool1
adipose_tissue,_adult,_pool1
esophagus,_adult,_pool1
bladder,_adult,_pool1
prostate,_adult,_pool1
ovary,_adult,_pool1
uterus,_adult,_pool1
liver,_adult,_pool1
pancreas,_adult,_donor1
CAGE TPM
b
HPA sample expressions (protein coding)
RNA−Seq FPKM
c
FANTOM5 sample expressions (non−coding)
CAGE TPM
d
HPA sample expressions (non−coding)
RNA−Seq FPKM

Figure S2a

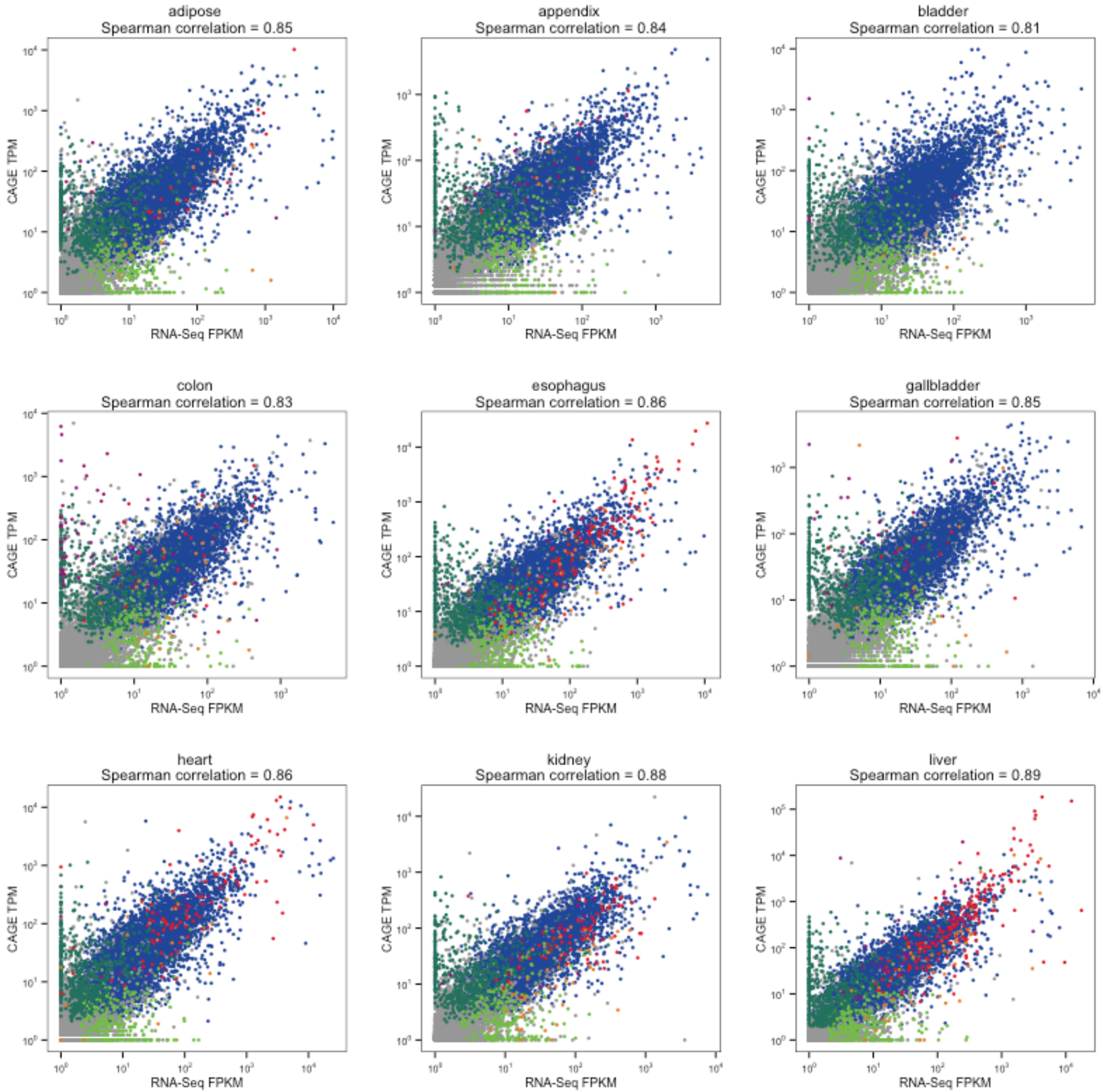
adipose
Spearman correlation = 0.85
appendix
Spearman correlation = 0.84
bladder
Spearman correlation = 0.81
colon
Spearman correlation = 0.83
esophagus
Spearman correlation = 0.86
gallbladder
Spearman correlation = 0.85
heart
Spearman correlation = 0.86
kidney
Spearman correlation = 0.88
liver
Spearman correlation = 0.89
CAGE TPM
RNA-Seq FPKM

Figure S2b

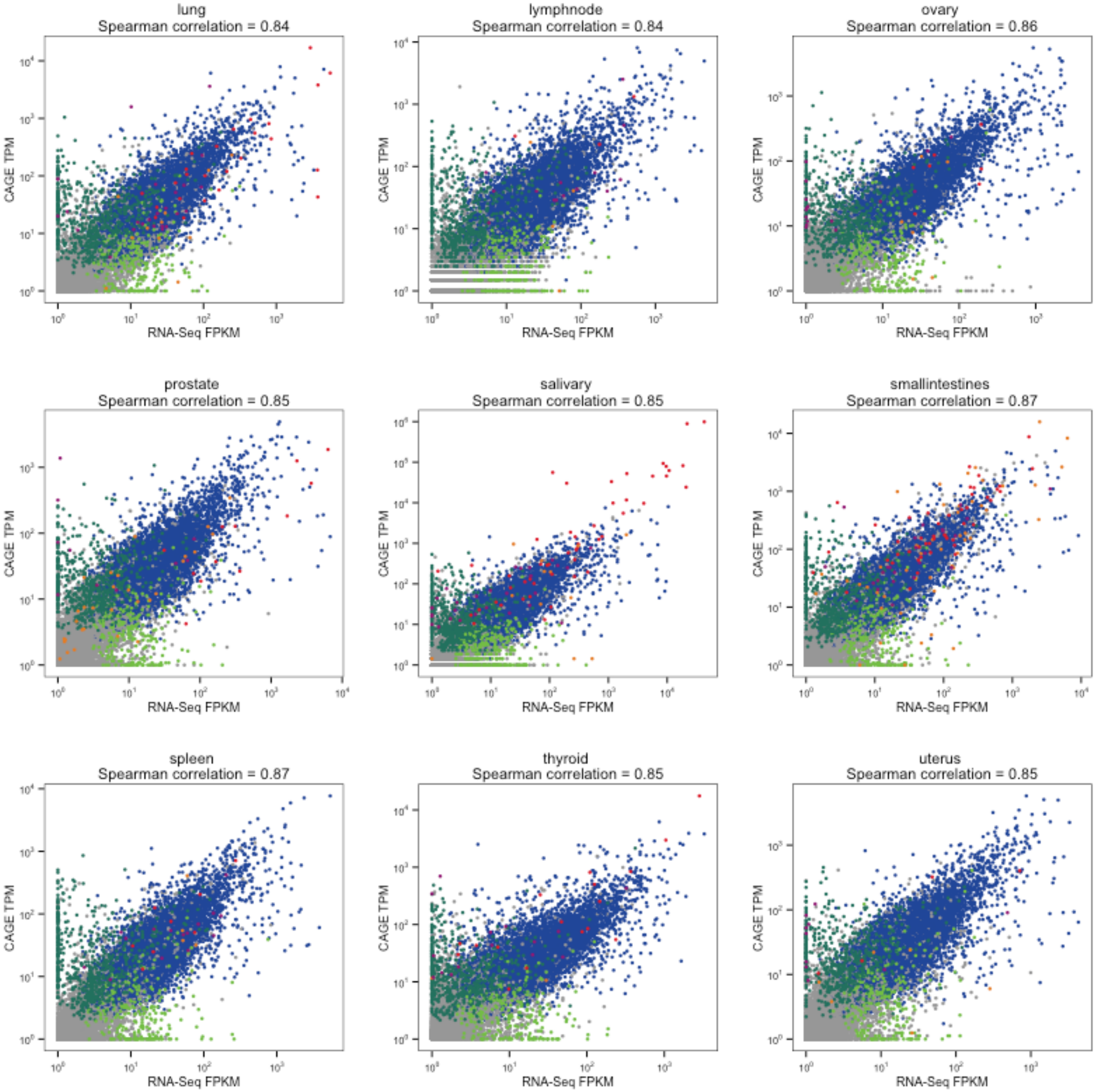
lung
Spearman correlation = 0.84
lymphnode
Spearman correlation = 0.84
ovary
Spearman correlation = 0.86
prostate
Spearman correlation = 0.85
salivary
Spearman correlation = 0.85
smallintestines
Spearman correlation = 0.87
spleen
Spearman correlation = 0.87
thyroid
Spearman correlation = 0.85
uterus
Spearman correlation = 0.85
CAGE TPM
RNA-Seq FPKM

Figure S2 legend

- Expressed in all tissues, CAGE and RNAseq
- Expressed in all tissues, CAGE only
- Expressed in all tissues, RNAseq only
- Tissue enriched, CAGE and RNAseq
- Tissue enriched, CAGE only
- Tissue enriched, RNAseq only
- Other

Figure S3

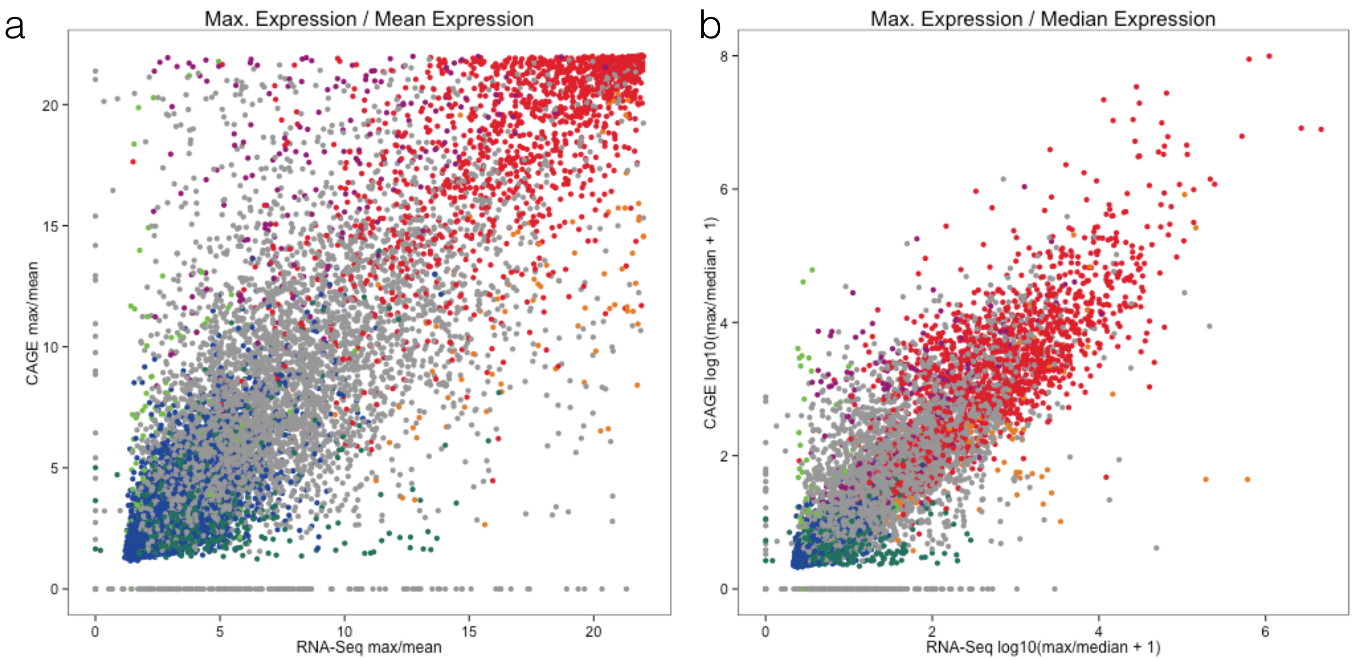

- Expressed in all tissues, CAGE and RNAseq
- Expressed in all tissues, CAGE only
- Expressed in all tissues, RNAseq only
- Tissue enriched, CAGE and RNAseq
- Tissue enriched, CAGE only
- Tissue enriched, RNAseq only
- Other

Figure S4

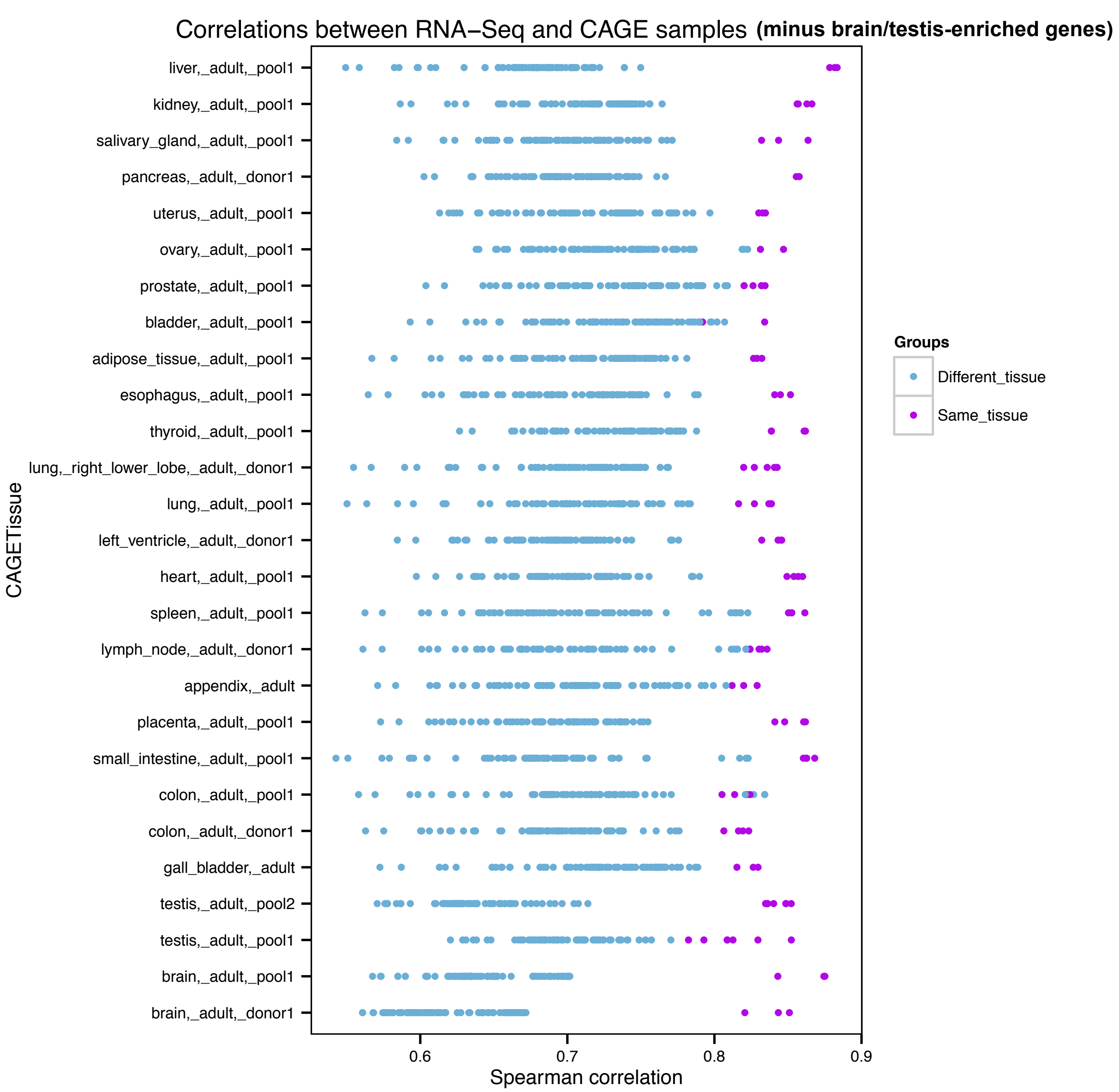

Correlations between RNA−Seq and CAGE samples (minus brain/testis-enriched genes)
liver,_adult,_pool1
kidney,_adult,_pool1
salivary_gland,_adult,_pool1
pancreas,_adult,_donor1
uterus,_adult,_pool1
ovary,_adult,_pool1
prostate,_adult,_pool1
bladder,_adult,_pool1
adipose_tissue,_adult,_pool1
esophagus,_adult,_pool1
thyroid,_adult,_pool1
lung,_right_lower_lobe,_adult,_donor1
lung,_adult,_pool1
left_ventricle,_adult,_donor1
heart,_adult,_pool1
spleen,_adult,_pool1
lymph_node,_adult,_donor1
appendix,_adult
placenta,_adult,_pool1
small_intestine,_adult,_pool1
colon,_adult,_pool1
colon,_adult,_donor1
gall_bladder,_adult
testis,_adult,_pool2
testis,_adult,_pool1
brain,_adult,_pool1
brain,_adult,_donor1
CAGETissue
0.6
0.7
0.8
0.9
Spearman correlation
Groups
Different_tissue
Same_tissue

Figure S5

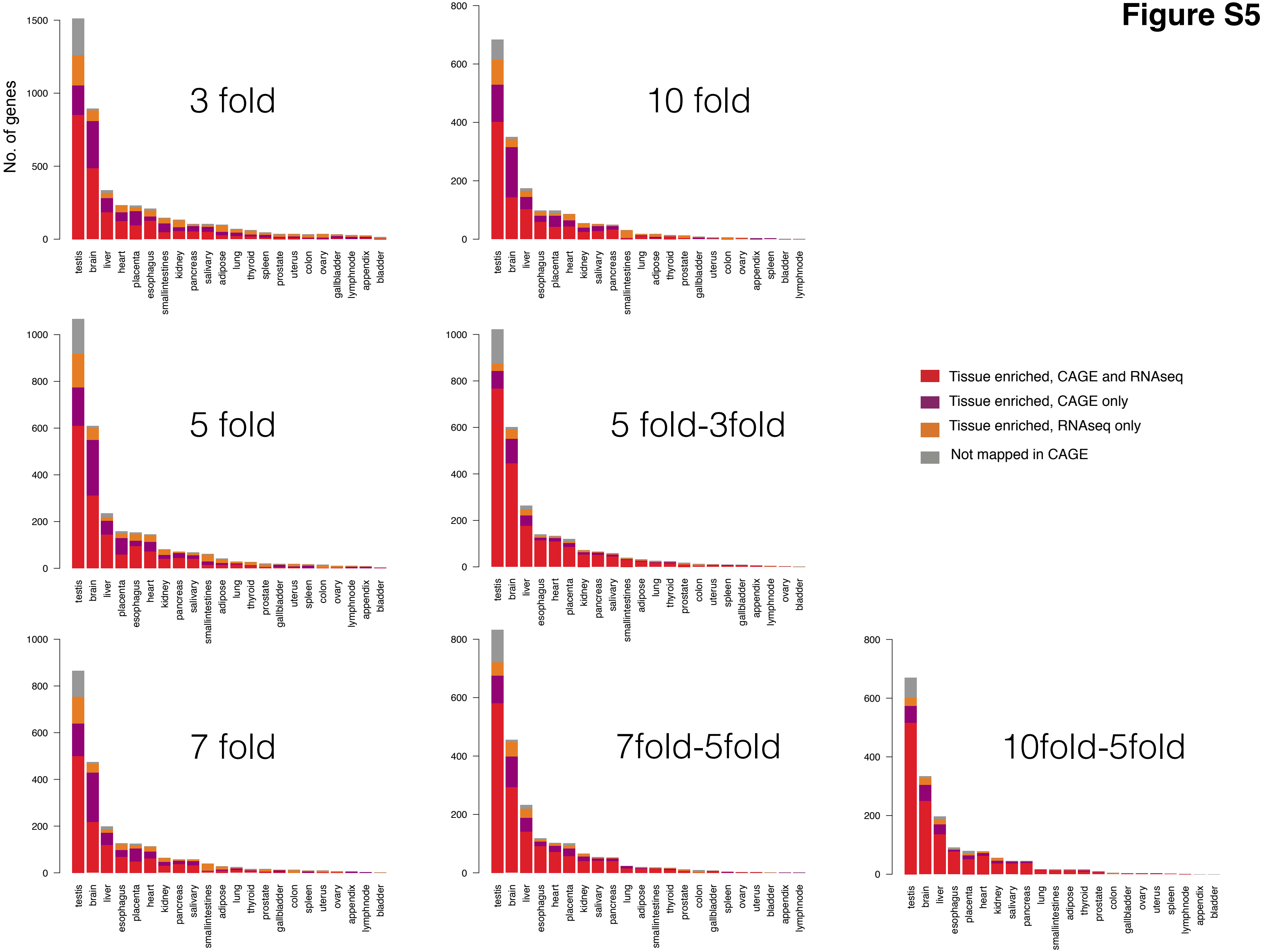

No. of genes
3 fold
10 fold
5 fold
5 fold-3fold
7 fold
7fold-5fold
10fold-5fold
Tissue enriched, CAGE and RNAseq
Tissue enriched, CAGE only
Tissue enriched, RNAseq only
Not mapped in CAGE
testis
brain
liver
heart
placenta
esophagus
smallintestines
kidney
pancreas
salivary
adipose
lung
thyroid
spleen
prostate
uterus
colon
ovary
gallbladder
lymphnode
appendix
bladder

Figure S6

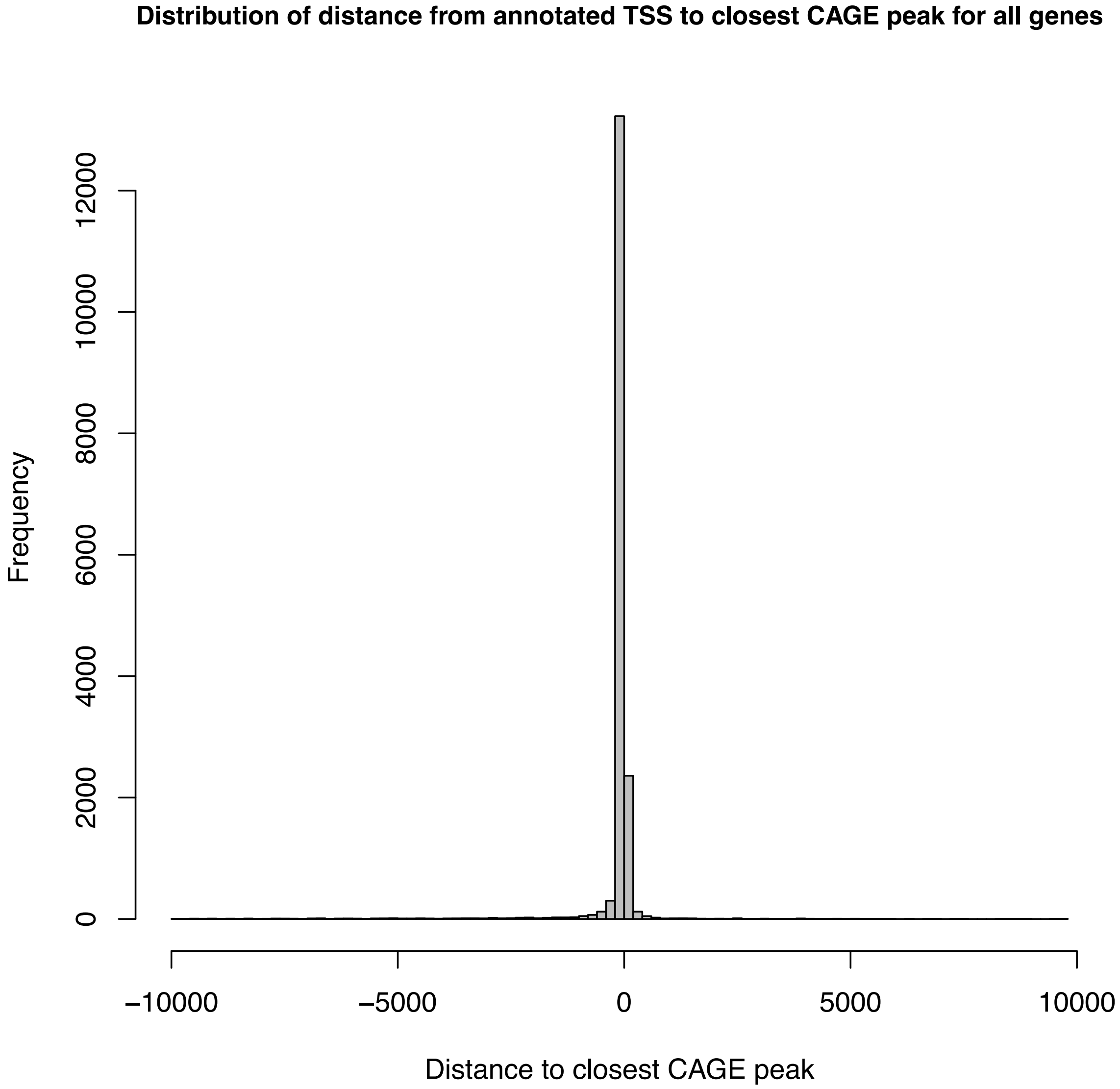
Distribution of distance from annotated TSS to closest CAGE peak for all genes
Frequency
0
2000
4000
6000
8000
10000
12000
−10000
−5000
0
5000
10000
Distance to closest CAGE peak

Figure S7

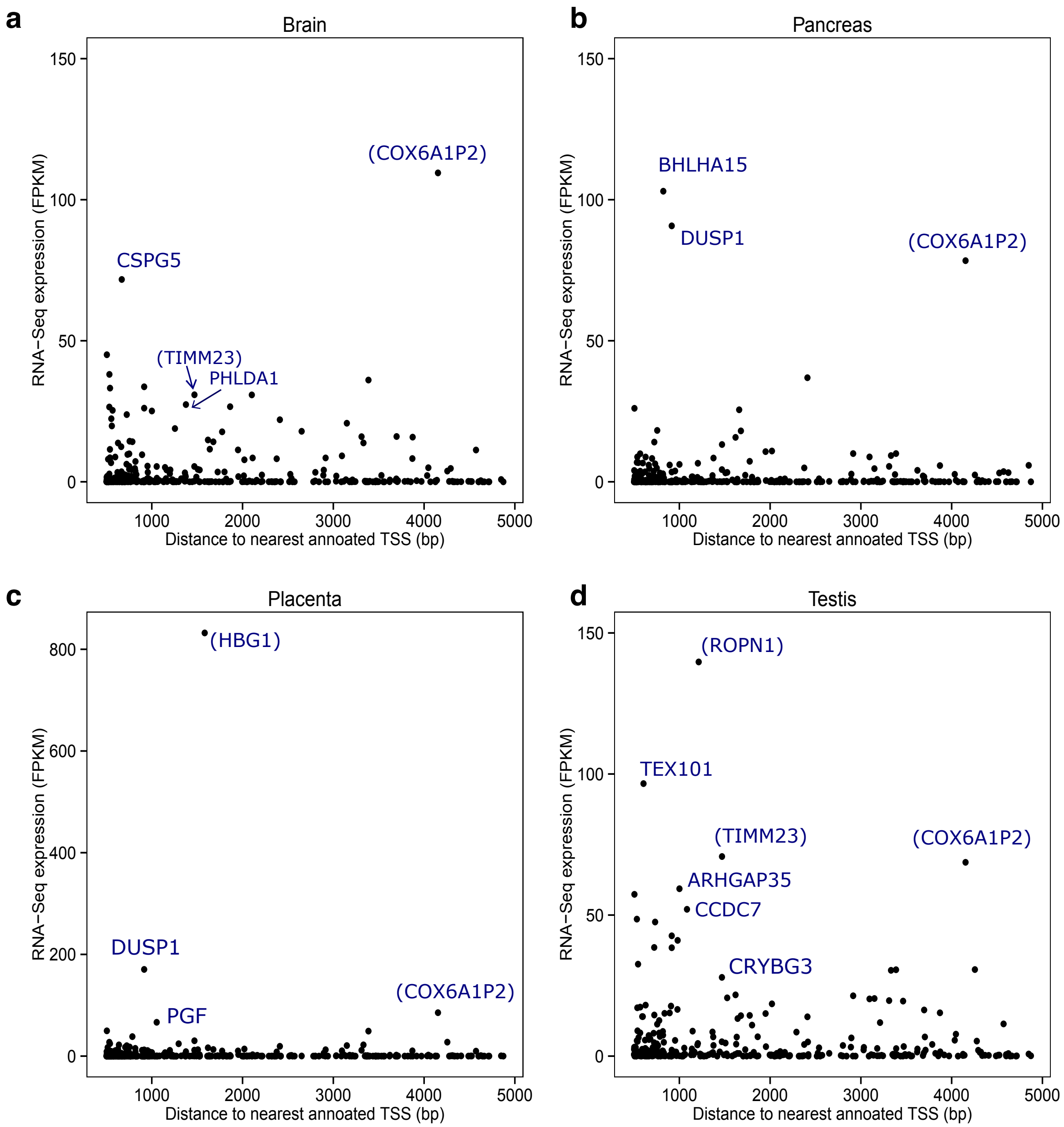

a
Brain
(COX6A1P2)
CSPG5
(TIMM23)
PHLDA1
b
Pancreas
BHLHA15
DUSP1
(COX6A1P2)
c
Placenta
(HBG1)
DUSP1
PGF
(COX6A1P2)
d
Testis
(ROPN1)
TEX101
(TIMM23)
(COX6A1P2)
ARHGAP35
CCDC7
CRYBG3
RNA−Seq expression (FPKM)
Distance to nearest annotated TSS (bp)

Figure S8

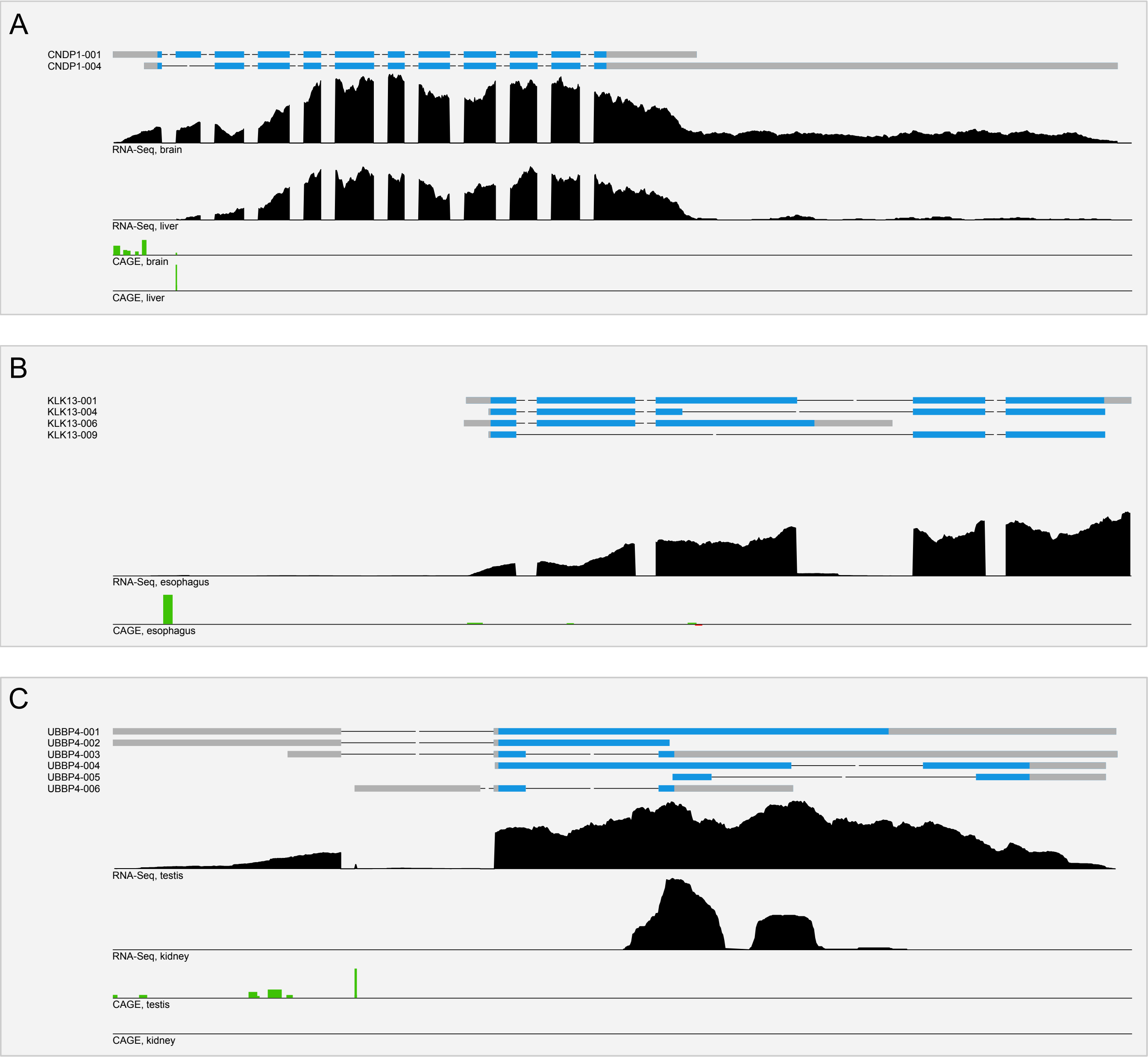

A
CNDP1-001
CNDP1-004
RNA-Seq, brain
RNA-Seq, liver
CAGE, brain
CAGE, liver
B
KLK13-001
KLK13-004
KLK13-006
KLK13-009
RNA-Seq, esophagus
CAGE, esophagus
C
UBBP4-001
UBBP4-002
UBBP4-003
UBBP4-004
UBBP4-005
UBBP4-006
RNA-Seq, testis
RNA-Seq, kidney
CAGE, testis
CAGE, kidney
